# Behavioral and Attitudinal Correlates of Trusted Sources of COVID-19 Vaccine Information in the US

**DOI:** 10.3390/bs11040056

**Published:** 2021-04-20

**Authors:** Carl A. Latkin, Lauren Dayton, Jacob R. Miller, Grace Yi, Afareen Jaleel, Chikaodinaka C. Nwosu, Cui Yang, Oluwaseun Falade-Nwulia

**Affiliations:** 1 Department of Health, Behavior and Society, Bloomberg School of Public Health, Johns Hopkins University, 615 N. Wolfe St., Baltimore, MD 21205, USA; ldayton2@jhu.edu (L.D.); gracetyi95@gmail.com (G.Y.); cyang29@jhu.edu (C.Y.); 2 Division of Infectious Diseases, Johns Hopkins University School of Medicine, 733 N. Broadway, Baltimore, MD 21205, USA; ofalade1@jhmi.edu; 3 Department of Epidemiology, Bloomberg School of Public Health, Johns Hopkins University, 615 N. Wolfe St., Baltimore, MD 21205, USA; jmill342@jhmi.edu; 4 Institute for Computational Medicine, Johns Hopkins University, 3400 N. Charles St., Baltimore, MD 21218, USA; ajaleel3@jhu.edu (A.J.); cnwosu3@jhu.edu (C.C.N.)

**Keywords:** COVID-19, SARS-CoV-2, vaccine, trust, information sources, health behaviors, health disparities

## Abstract

There is a critical need for the public to have trusted sources of vaccine information. A longitudinal online study assessed trust in COVID-19 vaccine information from 10 sources. A factor analysis for data reduction revealed two factors. The first factor contained politically conservative sources (PCS) of information. The second factor included eight news sources representing mainstream sources (MS). Multivariable logistic regression models were used. Trust in Dr. Fauci was also examined. High trust in MS was associated with intention to encourage family members to get COVID-19 vaccines, altruistic beliefs that more vulnerable people should have vaccine priority, and belief that racial minorities with higher rates of COVID-19 deaths should have priority. High trust in PCS was associated with intention to discourage friends from getting vaccinated. Higher trust in PCS was also associated with participants more likely to disagree that minorities with higher rates of COVID-19 deaths should have priority for a vaccine. High trust in Dr. Fauci as a source of COVID-19 vaccine information was associated with factors similar to high trust in MS. Fair, equitable, and transparent access and distribution are essential to ensure trust in public health systems’ abilities to serve the population.

## 1. Background

The COVID-19 pandemic has had a profound impact on global mental and physical health [1,2,3,4]. Adequate access to and uptake of COVID-19 vaccines are essential to curbing the COVID-19 pandemic. There has, however, been a plethora of COVID-19 misinformation [5,6,7], and concerns have been raised about the trustworthiness of sources of COVID-19 information [8]. Trust is a crucial factor in vaccine acceptance and hence may be critical for COVID-19 vaccine uptake [9,10]. Moreover, perceptions of the trustworthiness of health information can have a significant impact on health behaviors [10,11,12,13].

Trust has previously been identified as a key variable in vaccine decision making, and trust in health information has been conceptualized in various ways throughout existing literature [14,15,16]. Cairns, de Andrade, and MacDonald conceptualize trust as, in part, a cognitive tool to evaluate information [11]. Trust in sources of information can consequently facilitate decision making. In situations with limited, inconsistent, and contradictory information, trust may help foster receptivity to information. Decisions on whether to trust and in whom to trust are also critical, even more so when there is misinformation.

To make informed decisions about vaccine uptake, it is imperative that decision makers have trusted sources of information. The 2015 US National Vaccine Advisory Committee describes multiple avenues of trust, distinguishing between “the trust that parents or healthcare providers have in (1) the recommended immunizations, (2) the provider(s) who administers vaccines, and (3) the process that leads to vaccine licensure and the recommended vaccination schedule [17]”. The unprecedented and expedited process of developing and approving COVID-19 vaccines may significantly impact COVID-19 vaccine trust.

Vaccine information may come from several outlets, such as government sources, scientific organizations, individuals, mainstream and social media, and peers. On social media in particular, a vast amount of COVID-19 vaccine misinformation has been linked to vaccine hesitancy [18,19,20,21,22]. Given the compounding effects of widespread misinformation on the magnitude of the pandemic and effective prevention measures, as well as varying levels of risk among different populations and geographic areas, understanding trust in sources of COVID-19 information is particularly important. In addition, the promotion of several vaccines, with variations in dosing schedule, side effects, and efficacy, may lead to information overload. Trusted information sources may ameliorate information overload by helping individuals prioritize information and weed out dubious claims [23].

## 2. Literature Review 

Few studies have assessed trust in news sources during the COVID-19 pandemic. A study of sources of COVID-19 information by Ali et al. reported that the most frequently used sources of information were from the government and doctors, followed by TV and social media [24]. The two sources listed as most trusted for COVID-19 information by a substantial number of participants were the government (43%) and doctors (30%) [24]. Infrequently listed were newspapers (6%), websites (5%), and TV (5%) [24]. Social media was listed by only 1% of respondents as one of the most trusted sources of COVID-19 information [24]. A second study in the US conducted by Fridman et al. found that high trust in government sources of COVID-19 information was associated with accurate COVID-19 knowledge and participation in social distancing [25]. However, trust in social media sources such as Facebook or Twitter was negatively associated with accurate knowledge and participation in social distancing [25]. Many Americans have also expressed dissatisfaction with media coverage of the pandemic, with a Pew 2020 Research Center poll reporting that only approximately half (54%) of Americans thought that media coverage of COVID-19 was excellent or good [26].

The current study examined how trusted sources of COVID-19 vaccine information group together. Trust in vaccines is often conceptualized as unidirectional, with sources of trust influencing vaccine recipients. However, potential vaccine recipients can also influence others [27]. Consequently, in this study, we also examined how levels of trust in different sources of vaccine information may lead to encouraging or dissuading others to become vaccinated. 

Several other factors have been shown to influence vaccine trust and uptake. Indeed, Larson et al. [9] speculated that vaccine uptake is influenced by trust in vaccine-makers, such as pharmaceutical companies. In the United States, pharmaceutical companies are among the most poorly regarded industries [28]. This perception may be partly due to the pharmaceutical industry’s role in the ongoing opioid epidemic, where they have employed aggressive marketing tactics, exaggerated benefits, downplayed risks, and failed to warn the public of the addictive nature of opioids [29,30].

Political affiliation is another factor that may influence vaccine trust [26,31]. In the US and other countries, there has been a significant partisan divide in the opinions and coverage of the COVID-19 pandemic. During the early stages of the pandemic, right-leaning media outlets were more likely than mainstream outlets to promulgate COVID-19 misinformation [32]. In turn, misinformed people were more likely to believe that the CDC had exaggerated COVID-19 health risks [32]. Some research also suggests that conservatives are less likely to engage in COVID-19 prevention behaviors [33].

Social network diffusion is further theorized to affect vaccine trust and uptake. Due to high rates of COVID-19 vaccine hesitancy and distrust in information sources, social network diffusion of positive COVID-19 vaccine perceptions may be important in promoting uptake of COVID-19 vaccines. Several studies have previously examined social norms and vaccine attitudes, though much of this research has centered on human papillomavirus (HPV) vaccines [34,35,36]. Previous research on social norms also tends to focus on how norms influence individuals, primarily assessing study respondents’ own behaviors and attitudes. In the current study, we were interested in whether trust in COVID-19 vaccine information sources was associated with respondents’ intentions to promote COVID-19 vaccination among their social networks. We also anticipated that individuals with lower trust in COVID-19 vaccine information from credible mainstream sources would dissuade their peers from becoming vaccinated. 

Finally, there have been major health disparities in the COVID-19 pandemic, with Black and Hispanic populations experiencing higher rates of SARS-CoV-2 infection, hospitalization, and mortality compared with White populations [37,38]. This trend has been well documented and widely reported, as have high mortality rates among older Americans. Given these health disparities, it is important to ensure equity in vaccine access and support for addressing COVID-19 health disparities. Consequently, we assessed whether there was an association between the level of trust in sources of COVID-19 vaccine information and attitudes toward prioritizing older Americans and prioritizing Black and Hispanic populations. 

This study aimed to examine underlying dimensions of trust in COVID-19 vaccine information sources. We first evaluated how trust in certain sources of information correlated with trust in other information sources. We then examined whether trust in various sources of COVID-19 vaccine information was linked to vaccine intentions. Next, we assessed sociodemographic and behavioral factors associated with trust in sources of COVID-19 vaccine information. We hypothesized that trust in COVID-19 vaccine information would be associated with political ideology, with less trust among conservatives in mainstream sources of COVID-19 vaccine information. We also anticipated that trust in sources of COVID-19 information would be associated with greater concerns about side effects and vaccine effectiveness. Contrary to traditional unidirectional models in the literature, our model of vaccine trust was conceptualized to be bidirectional, with vaccine concerns leading to lower levels of trust in COVID-19 information and, conversely, lower levels of trust in COVID-19 information also leading to greater vaccine concerns. We further assessed how trust in COVID-19 vaccine information might be linked to perceptions of vaccine priorities for groups with high rates of COVID-19 mortality, such as older individuals and certain racial and ethnic minorities. Finally, we examined factors associated with trust in COVID-19 information from Dr. Anthony Fauci, Director of the National Institute of Allergies and Infectious Diseases, who has been a prominent scientist in the US response to the COVID-19 pandemic, a member of the White House COVID-19 task force, and a frequent public commentator on the pandemic and vaccine efforts. 

## 3. Methods

Study respondents participated in an online four-wave longitudinal study that began in March 2020. This study aimed to examine individual, social, and societal-level fluctuations amid the rapidly-changing landscape of the pandemic. Study periods occurred every few months and aimed to capture changes in scientific knowledge of infection, extent of infectious spread, and progress in vaccine development. The fourth wave of this study was administered from 18 to 28 November 2020, which was after Pfizer-BioNTech (9 November, Kalamazoo, MI, USA) and Moderna (16 November, Norwood, MA, USA) presented preliminary Phase 3 data indicating that their COVID-19 vaccines were more than 90% effective. The baseline sample was 809. There were 586 valid surveys at wave four and 522 individuals who completed both wave three and wave four surveys.

Study participants were recruited through Amazon’s Mechanical Turk (MTurk, Amazon, Seattle, WA, USA), which is a commercially available service commonly used for surveys and other types of academic research. This approach is regularly used by health researchers, as it allows for a diverse sample to be collected in a rapid and timely fashion [39]. As research has indicated, MTurk provides better-quality data in less time than other methods for recruiting convenience samples [40]. Study populations recruited through MTurk are not nationally representative, but have been documented to outperform other opinion samples on several dimensions (such as attracting populations like young individuals interested in news, Hispanic and Asian respondents, and individuals from industries and geographic locations that parallel national, professionally-collected samples) [41]. Studies using MTurk have also demonstrated good reliability [42]. The study protocols followed MTurk’s best practices, which included ensuring participant confidentiality, protecting study integrity, generating unique completion codes, integrating attention checks throughout the survey, repeating study-specific qualification questions, and removing ineligible participants [40,43,44]. Moreover, despite COVID-19, the demographic characteristics of MTurk appear to be stable [45]. Eligibility included being age 18 or older, living in the United States, being able to speak and read English, having heard of the coronavirus or COVID-19, and providing written informed consent. Additionally, to enhance reliability, eligible participants had to pass attention and validity checks embedded in the survey [46]. Following recommendations by Rouse and colleagues [46], we embedded checks to mitigate inattentive and random responding. These checks included survey questions with exceedingly low probabilities, such as deep-sea fishing in Alaska and having appendages removed. We also repeated questions to ensure consistency. Finally, we examined the amount of time participants took to complete the survey and verified the completeness of the data. Participants were compensated $2.50 for completing the first survey, $3.00 for the second, $3.50 for the third, and $4.00 for the fourth, which was equivalent to approximately $12 per hour. The study protocols were approved by the Johns Hopkins Bloomberg School of Public Health Institutional Review Board. 

To assess trust in sources of COVID-19 vaccine information, a set of questions asked participants, “How much do you trust information about the vaccine for coronavirus from the following sources?” (1) the CDC, (2) the White House, (3) Johns Hopkins University, (4) CNN, (5) Fox News, (6) your State Health Department, (7) your healthcare provider, (8) Anthony Fauci, Director of the National Institute of Allergy and Infectious Diseases, (9) pharmaceutical or drug companies, and (10) the US Food and Drug Administration (FDA). Response options were “(4) A great deal”, “(3) Quite a bit”, “(2) Some” and “(1) Very little or none”. These sources were chosen based on survey items used in US national polls or prior studies on vaccine trust [47,48], as well as on information sources anticipated to provide accurate information [14]. Johns Hopkins University was chosen due to its major role in disseminating COVID-19 data, and Anthony Fauci was selected for his lead role in addressing the pandemic in the US and serving as a spokesperson for the scientific community.

Items also assessed COVID-19 vaccine attitudes and behavioral diffusion intentions. Three questions assessed attitudes regarding vaccine concerns: “I am concerned that the coronavirus vaccines are being developed too quickly”, “I am worried about having bad side effects if I got a coronavirus vaccine” and “I am concerned that a coronavirus vaccine will not be effective”. COVID-19 vaccine attitudes were assessed with the question: “A vaccine would prevent me from getting the coronavirus”. Altruistic attitudes were assessed with the items: “More vulnerable people such as the elderly should have priority for a coronavirus vaccine” and “Groups that have higher rates of coronavirus deaths, such as Blacks and Latinos, should have priority for a coronavirus vaccine”. Intention to diffuse COVID-19 vaccination norms was assessed with the questions: “I will discourage my friends from getting a coronavirus vaccine when it is available” and “I will encourage my family members to get a coronavirus vaccine when it is available”. COVID-19 vaccine intentions were measured with the item: “I am very likely to get a coronavirus vaccine, when available”. The response options to these questions were “strongly agree”, “agree”, “neither agree nor disagree”, “disagree” and “strongly disagree”. 

The choices for race/ethnicity were “White”, “Non-Hispanic Black”, “Hispanic”, “Asian”, “Mixed” and “Other”. Due to small sample sizes, “Mixed”, “Hispanic” and “Other” categories were collapsed into one category. Biological sex, age, education, and income were also assessed. Level of education was dichotomized at bachelor’s degree or higher. Income was dichotomized at $60,000 or below. Political party affiliation was assessed with the standard question, “Do you consider yourself Republican, Democrat, Independent, Libertarian or other?” Due to the small cell size, Libertarian and “Other” groups were collapsed into one “Other” group. 

## 4. Analyses

**Figure 1:**
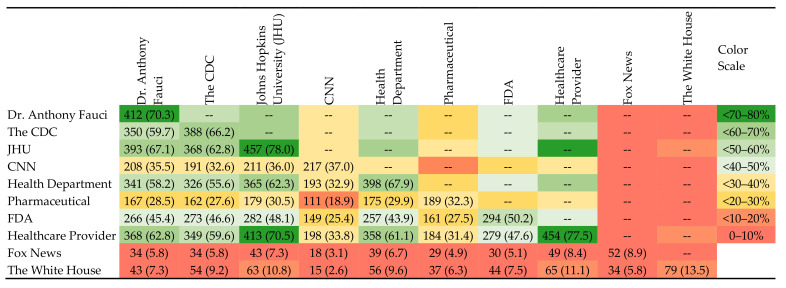
Concordance among high trust ratings of 10 sources of COVID-19 vaccine information among study participants in the US, 2020, *N* (%).

We examined both individual informational trust variables as well as aggregate measures of trust. Descriptive statistics for the sample (*N* = 586) were used to characterize the distribution of trust of the ten information sources. Responses to trust in information sources were dichotomized as high (a great deal or quite a bit) versus low (some, very little or none). We then examined the relationships among the items by constructing a 10 × 10 matrix of high trust among the information sources (Figure 1). To reduce the number of dependent variables, we conducted a factor analysis. We used the principal component method with the ten vaccine informational trust items. This analysis identified a two-factor structure, and two scales were formed, one with 2 items and one with 8 items. The 2-item scale contained politically conservative sources (PCS) of information, including Fox News and the White House. The 8-item scale contained news sources representing mainstream sources (MS). For the scales, the items within each scale were added together. 

We also used an analysis of variance (ANOVA) test to examine the relationship between vaccine intentions and trust in the COVID-19 vaccine information sources. Informational trust was the dependent variable, as measured by the MS and PCS scales of trust in COVID-19 vaccine information. We employed a three-group ([1] strongly agree, agree, [2] neither agree or disagree, and [3] disagree, strongly disagree) ANOVA with post hoc comparisons among the positive, neutral, and negative vaccine intention groups. 

To measure independent associations between trust in the vaccine and vaccine intentions, we constructed multiple multivariable regression models. The two scales on trust in sources of COVID-19 information were treated as dependent variables, and OLS linear and logistic regression models were built for the MS scale to examine both dichotomous (logistic) and continuous (linear) associations. Logistic regression models for the 2-item PCS scale were also employed, but an OLS regression model was not feasible for this scale. A median split was used to dichotomize the 8-item MS scale for the logistic regression model. An additional logistic regression model examined predictors of level of COVID-19 vaccine trust in information from Dr. Anthony Fauci, director of NIAID. A single participant who was missing data on the dependent variable was excluded from the analysis for a final sample size of *N* = 585 for the regression models. These regression models included demographic factors and variables hypothesized to be related to trust in information about COVID-19 vaccines, which include risk perceptions of COVID-19 as well as views of priorities about vulnerable groups and encouraging vaccination among family and friends. For the multivariable logistic regression models, all sociodemographic variables and other variables with a *p*-value of <0.20 in the bivariate models were included in the final adjusted model [49,50].

## 5. Results 

Sample demographics (*N* = 586) are presented in Table 1. 

Just under half of the sample reported Democratic political affiliation (45.7%), with 21.2% Republican, 29.0% Independent, and the remaining 4.1% reporting “Other”. The majority of the sample was White (81.1%), with 6.3% reporting Black and 6.7% reporting Asian. Due to the small number of Hispanic respondents, Hispanic participants were collapsed with the “Other race/ethnicity” category, which in total comprised 6.0% of respondents. The mean age of the participants was 39.6 (SD 11.7). Approximately half of the sample (57.3%) was female, 55.7% had a bachelor’s degree or higher, and 45.6% had a household income greater than $60,000.

Descriptive statistics for vaccine attitudes, behaviors, and news consumption are reported in Table 2. 

Under half of the sample (60.9%) agreed or strongly agreed that a vaccine would prevent infection with the coronavirus, while 12.1% disagreed or strongly disagreed. The majority of participants (85.4%) disagreed or strongly disagreed with the statement that they would “discourage [their] friends from getting the coronavirus vaccine when available”. Conversely, a smaller proportion (59.4%) agreed or strongly agreed that they would encourage family members to get a vaccine when available, with 19.1% disagreeing or strongly disagreeing. More than half of the sample (64.0%) reported worry about bad side effects, with 47.1% reporting concern that a vaccine would not be effective and 56.8% reporting concern that short cuts had been taken with the vaccine due to political pressures. The majority of the sample agreed or strongly agreed that more vulnerable people should have priority for a coronavirus vaccine (86.9%) and that groups with higher rates of coronavirus deaths should have priority for a vaccine (66.0%). One-third of participants (35.2%) reported watching, listening, or reading news about the coronavirus “a couple times a day”, with 29.6% reporting “once a day”, 21.5% reporting “less than once a day”, 9.9% reporting “every 1–2 h” and 3.8% reporting “multiple times an hour”. 

The factorability of the 10 information sources was examined using principal component analyses for data reduction.

As shown in Table 3, based on the scree plot and Kaiser’s criterion of eigenvalues greater than one, we arrived at a two-factor solution that explained 66.3% of the variance for the entire set of variables. The Kaiser–Meyer–Olkin Measure of Sampling Adequacy was 0.87, with a chi-square value of 3077.85, df = 45, *p* = 0.000. Factor 1 contained eight items and was labeled “Mainstream Sources (MS)”, as these items represented sources acknowledged for promoting scientifically credible information. This factor explained 48% of the variance. Factor 2 contained two items that represented politically conservative sources and was accordingly labeled “Politically Conservative Sources (PCS)”. This factor explained 18% of the variance. Factors 1 and 2 had eigenvalues of 4.84 and 1.79, respectively. For the 8-item MS scale, the Cronbach’s alpha was 0.91, and removing any of the items did not change the Cronbach’s alpha more than 0.02. The corrected item correlations ranged from 0.58 to 0.78, with most in the mid 0.7 range, suggesting a scale with robust properties. Overall, analyses indicated that there were two distinct factors underlying trust in COVID-19 vaccine information sources and that these factors were internally consistent. Trust scores for the 2-item PCS scale ranged from 2 to 8 (mean 7.04, median 8.0) and were dichotomized at 6, which translates to “some” or “a great deal” of trust in both sources or “quite a bit” of trust in one of the two sources. After the scale was dichotomized, 26.3% of the respondents were in the higher trust category. For the 8-item MS scale, values ranged from 8 to 32 (mean 18.12, median 17.0). A median split was used to dichotomize the 8-item MS scale for the logistic regression model.

Out of 10 potential news sources, participants had both a mean and median of five high-trust sources of COVID-19 vaccine information. The highest levels of trust were reported to be in Johns Hopkins University (78.0%), healthcare providers (75.5%), Dr. Fauci (70.3%), state health departments (67.9%), and the CDC (66.2%), while the lowest levels of trust were reported to be in the White House (13.7%) and Fox News (8.9%) (Table 3). Half of the sample had high trust in four or more of the sources on the MS scale, while 16% had high trust in one or two of the sources on the 2-item PCS scale. Only 10% of the respondents reported not having high trust in any of the ten sources. 66.8% of respondents listed high levels of trust in one or more of the items on the 8-items MS scale, and only 4.6% reported high levels of trust in one or two items on the 2-item PCS scale.

Figure 1 presents concordance analyses displaying relationships between high trust levels among various sources of COVID-19 vaccine information. The figures listed in each box represent the percentage of the total sample (*N* = 586) who reported high trust in both corresponding sources. For example, 59.7% reported high trust in both the CDC and Dr. Fauci as sources of COVID-19 vaccine information. Among the items on the 8-item MS scale, there were high levels of high trust concordance (~60–70%) between trust in Dr. Fauci, the CDC, Johns Hopkins University (JHU), state health departments, and healthcare providers. Lower levels of concordance were noted between these same sources and CNN (~35%), pharmaceutical companies (~30%), and the FDA (~45%). The majority of those with high trust in Fox News had low trust (≤10% concordance) in Dr. Fauci, the CDC, JHU, CNN, the health department, pharmaceutical companies, the FDA, healthcare providers, and the White House. Similarly, there were low levels of concordance with trust in the White House and all other sources (≤14%). Among those with high trust in healthcare providers, very few also had high trust in Fox News (8.4%) or the White House (11.1%). Regardless of levels of trust in other sources, most of the respondents (>70%) reported high trust in Dr. Fauci, JHU, and healthcare providers. A fixed-effects one-way ANOVA was conducted to compare the effect of trust in MS and PCS, respectively, on the perceived likelihood of getting the coronavirus vaccine, in positive, neutral, and negative intention conditions (Table 4). 

For the 8-item MS scale, there was a significant effect of trust in mainstream sources on coronavirus vaccine intention at the *p* < 0.05 level for the three conditions [F(2, 582) = 71.2, *p* < 0.001]. Conversely, for the 2-item PCS scale, there was no significant effect of trust in politically conservative sources on coronavirus vaccine intention [F(2, 582) = 0.29, *p* = 0.75]. 

As reported in Table 5, post hoc comparisons using the Tukey HSD test revealed significant differences in trust between the neutral and negative groups (1.12 ± 0.26, *p* < 0.001), between positive and negative groups (2.32 ± 0.20, *p* < 0.001), and between positive and neutral groups (1.20 ± 0.26, *p* < 0.001). 

Table 6 shows results from bivariate and multivariable logistic analyses with the binary 8-item MS scale as the dependent variable. As previously mentioned, the sample size used for regression analyses was *N* = 585 due to missing data on the dependent variable from one study participant. In bivariate analyses, covariates measuring vaccine concerns (side effects, efficacy, shortcuts), beliefs (vaccine would prevent infection, vulnerable people should have priority), and diffusion intentions (intention to encourage friends or family members to receive a vaccine) were significantly associated with trust in MS. In addition, bivariate analyses showed that, compared with Republican affiliation, Democratic affiliation was significantly and positively associated with trust in MS, while Libertarian or Independent affiliation was associated with lower odds of trust in MS. Bivariate analyses also indicated significant associations between trust in MS and greater educational attainment as well as greater news consumption about the coronavirus. In models adjusted for all covariates, including demographic factors, the majority of these associations remained robust. Trust in mainstream sources was significantly associated with belief that a vaccine could prevent coronavirus infection (aOR = 1.47, 95% CI = 1.10, 1.96). Trust in MS sources was also associated with the intention to encourage family members to get the COVID-19 vaccine (aOR = 1.61, 95% CI = 1.28, 2.04). Higher trust in MS sources was further associated with altruistic beliefs surrounding the vaccine, such as believing that more vulnerable people should have priority (aOR = 1.50, 95% CI = 1.09, 2.05) and that groups with higher rates of coronavirus deaths should have priority (aOR = 1.32, 95% CI = 1.05, 1.64). While higher trust in MS was associated with democratic political affiliation (aOR = 2.82, 95% CI = 1.59, 5.02), higher trust was not associated with other demographic factors such as race, gender, income, or education.

Results from bivariate and multivariable logistic analyses with the binary 2-item PCS measure as the dependent variable are also presented in Table 6. Similar to the associations between trust in MS and vaccine intentions, bivariate analyses revealed significant associations between trust in PCS and vaccine diffusion intentions, concerns surrounding shortcuts, altruistic beliefs, and political affiliation. In adjusted models, unlike those with high trust in MS, high trust in PCS was not associated with the belief that a vaccine would prevent coronavirus infection. Further, high trust in PCS was independently associated with intention to discourage friends from getting the vaccine when available (aOR = 1.47, 95% CI = 1.12, 1.91). Greater trust in PCS was also correlated with reduced concern about shortcuts with the COVID-19 vaccine due to political pressures (aOR = 0.70, 95% CI = 0.53, 0.85) and with reduced altruistic intention (aOR = 0.70, 95% CI = 0.56, 0.88). Political affiliation was associated with trust in PCS, with democrats (aOR = 0.11, 95% CI = 0.06, 0.20) and independents (aOR = 0.28, 95% CI = 0.17, 0.48) with significantly reduced trust in PCS. 

Bivariate and multivariable logistic analyses with trust in Dr. Fauci as the dependent variable, dichotomized as high vs. little to none, showed significant associations between trust in Dr. Fauci and COVID-19 vaccine diffusion intentions, as well as the belief that a vaccine would prevent infection (Table 6). In bivariate analyses, similar to the relationship between trust in MS and vaccine perceptions, trust in Dr. Fauci was also associated with decreased worry (about side effects, vaccine efficacy, and short cuts due to political pressures) and altruistic beliefs. Unlike previous associations, bivariate models further indicated associations between trust in Dr. Fauci and increased news consumption. Certain sociodemographic variables (“Other” race, greater educational attainment, and Democratic political affiliation) were also significant in bivariate analyses. In adjusted models, trust in Dr. Fauci was still independently associated with the belief that a vaccine would prevent infection with the COVID-19 (aOR = 1.49, 95% CI = 1.10, 2.03). As with trust in MS, trust in Dr. Fauci was further associated with willingness to promote vaccination within social networks (aOR = 1.68, 95% CI = 1.31, 2.16), reduced likelihood of discouraging friends from getting the vaccine (aOR = 0.67, 95% CI = 0.51, 0.89), and altruistic beliefs (aOR = 1.41, 95% CI = 1.11, 1.79). Those who self-identified as Democrats (aOR = 4.23, 95% CI = 2.25, 7.96) or independents (aOR = 3.10, 95% CI = 1.66, 5.78) were more likely to report higher trust in Dr. Fauci. In addition, individuals self-reporting as “Other” race, a category including Hispanic individuals, were significantly more likely than White respondents to have greater trust in Dr. Fauci (aOR = 5.65, 95% CI = 1.19, 26.9). 

When using the continuous 8-item MS measure as a dependent variable in linear regression models, for which higher scores indicate greater trust in MS, associations were nearly identical to those models that dichotomized the dependent variable representing trust in MS. As with trust in MS in the dichotomized measure, bivariate analyses indicated that trust in MS as a continuous measure was associated with diffusion intentions, reduced vaccine concerns, altruistic intentions, increased news consumption, and Democratic political affiliation (Table 7). These associations remained tenable in adjusted models, in which intention to discourage friends from getting the vaccine was negatively associated with trust in MS (aβ = −0.23, *p* < 0.05), while positive intention to encourage family members to get the vaccine was strongly associated with increased trust in MS (aβ = 0.36, *p* < 0.001). Altruistic beliefs were significantly and independently associated with increased trust in MS (aβ = 0.36, *p* < 0.01; aβ = 0.22, *p* < 0.01, respectively). Democratic political affiliation was also strongly associated with increased trust in MS (aβ = 0.60, *p* < 0.01). The residuals of the OLS model indicated that assumptions of a normal distribution were valid.

## 6. Discussion

The vast majority of study respondents reported high trust in at least one source of COVID-19 vaccine information. On average, participants reported high trust in five informational sources. It was promising that 89% of respondents reported high trust in at least one of the eight MS sources, and that 90% reported high trust in at least one of the ten sources. These findings suggest that many sources providing COVID-19 vaccine information to a large proportion of the sample are viewed as credible. Those who report higher levels of trust in mainstream sources also have more positive vaccine intentions. We do not know if individuals with lower levels of trust in COVID-19 vaccine information sources have a greater distrust of general vaccine information or greater distrust of mainstream sources. 

Although there was a high level of trust in healthcare providers for COVID-19 vaccine information compared to other sources, still more than 20% of respondents reported lower levels of trust in healthcare providers, suggesting that providers should not assume that all patients will view them as trustworthy sources of COVID-19 vaccine information. One approach for vaccine uptake campaigns could be to first ask individuals what information sources they trust from of a list of vetted sources, and then provide them COVID-19 vaccine information from that source.

We found that respondents who intended to become vaccinated reported higher trust in a greater number of informational sources than those who had negative vaccine intentions. On average, those with negative vaccine intentions reported two sources of COVID-19 vaccine information that they trusted “a great deal” or “quite a bit”. These findings suggest that there are trusted information channels that can be leveraged to address the concerns of individuals who do not intend to be vaccinated. However, although trust in vaccine information sources may be an important component in vaccine hesitancy, it is not the only component [27]. Given the strong correlations among the eight trusted MS sources of COVID-19 vaccine information, future research should examine whether these sources can work in consort, with some focusing on certain domains, such as vaccine effectiveness, or amplifying what the other seven sources report.

In examining varying levels of trust in sources of COVID-19 vaccine information, there were high intercorrelations from which two distinct scales emerged. Perhaps the most interesting finding was the preponderance of items *(N* = 8) that composed the MS scale. The finding indicates that scoring high on one of these eight items was correlated with scoring high on the others. The findings from the 8-item MS scale (comprised of the CDC, FDA, Johns Hopkins University, CNN, State Health Department, healthcare providers, Dr. Fauci, and pharmaceutical companies) indicated that those who reported a greater level of trust in COVID-19 information from the eight mainstream sources were more likely to encourage family members and less likely to discourage friends from being vaccinated. This association was more pronounced for the OLS regression than the logistic regression model, suggesting a linear trend. This finding highlights that trust in sources of health information may not only impact one’s own vaccine behaviors but also is likely to lead to individuals influencing others’ behaviors. This process is likely to inhibit vaccine uptake campaigns if those with low trust in credible information sources discourage others from becoming vaccinated and also are not receiving valid scientific information on the safety and efficacy of COVID-19 vaccines. 

A second scale, the 2-item PCS scale, was comprised of levels of trust in vaccine information from Fox News and the White House. In the regression models for the 8-item MS scale, those who scored higher on the scales were more likely to endorse the statement that vulnerable groups such as the elderly should have priority for the vaccine. For the 2-item PCS scale, the association was in the same direction, albeit not statistically significant (*p* = 0.10). In contrast, for the question on vaccine priority among groups with higher death rates, which explicitly listed Blacks and Latinos, individuals who scored high on the 8-item MS scale were significantly more likely to endorse the statement, whereas those respondents who scored high on the 2-item PCS scale were significantly less likely to endorse the statement. This finding is exceedingly troubling, as the main difference between the two questions on vaccine priorities, both of which referenced groups disproportionately affected by COVID-19, was that one mentioned specific racial and ethnic groups while the other mentioned the elderly. It appears that individuals who report high levels of trust in Fox News and/or the White House differentially judge vaccine priorities based on race/ethnicity versus age. This measure may capture racial biases when this survey question is used in conjunction with the question on priorities for older Americans or other vulnerable groups. In contrast to respondents who scored high on the 2-item PCS scale, individuals who scored high on the 8-item MS scale indicated that vulnerable populations should have vaccine priority based on both race/ethnicity and age. 

Not surprisingly, Democrats and Independents were less likely to score higher on the 2-item PCS scale. Democrats were more likely to score higher on the 8-item MS scale. These findings align with previous research on political party affiliation and trust in scientific information [51]. Prior research has also found that liberal and Democratic participants had more accurate COVID-19 information than conservative Republicans [52]. This same study found (1) that conservatives, compared to liberals, had less trust in scientific sources of COVID-19 information, (2) that individuals who reported CNN or Fox News as their main news source held less accurate knowledge than others, and (3) that accurate knowledge was associated with social distancing [53]. Another study reported that that COVID-19 vaccine hesitancy is associated with political beliefs [54]. These findings suggest that there is an intertwined relationship between political beliefs, news sources, COVID-19 knowledge, and COVID-19 behaviors. Individuals may seek information from sources with similar political orientations, which likely disseminate information that validates their personal beliefs and behaviors. This political polarization of COVID-19 information highlights the need to develop bipartisan sources of scientific information in responding to the COVID-19 pandemic. This approach would allow the dissemination of COVID-19 vaccine information that is explicitly endorsed by leaders of the major political parties. 

As with prior studies, healthcare providers were rated as highly trusted. Dr. Fauci was also rated as highly trusted. There have been historical precedents of public health figures playing a key role in the perception of public health issues, such as C. Everett Koop with both HIV and tobacco. It is likely that Dr. Fauci’s professional demeanor, his adherence to factual evidence, and his extensive media presence enhanced his influence. His high standing may also be due in part to his independence, as demonstrated by his stating positions counter to the Trump administration and his seeming indifference to the negative reactions of the Trump administration to his opinions. Being a white male with a non-upper-class accent may also be important attributes.

The high levels of COVID-19 vaccine information trust in Johns Hopkins University may be due to its frequently-cited web-based COVID-19 pandemic dashboard and data tracking, which is used by many news organizations. It is doubtful that most respondents seek information from the Johns Hopkins website; rather, it is more likely that they obtain information indirectly from mainstream and web-based news sources that quote Johns Hopkins. We did not assess other news sources or print media such as the New York Times that may also have high trust, due to lower viewership of such sources as compared to TV. However, it would be valuable to examine the relationship between additional sources of information and vaccine trust in future studies. Prior research suggests that individuals in the US obtain COVID-19 information from an average of six sources, most frequently from social media and websites [24]. However, when individuals are using the internet to obtain COVID-19 vaccine information, they may not know or remember the sources or websites, which suggests that it may be useful to collect real-time data on COVID-19 information-seeking in order to accurately assess the perceived credibility and validity of these sources. 

There was a relatively small group (17%) that reported high COVID-19 vaccine trust in at least one of the politically conservative news sources (i.e., White House, Fox News). These low numbers may be attributed to the political ideology of these two entities, to contextual factors such as the White House’s promotion of unproven treatments of COVID-19, or to both Fox News and the White House’s downplaying of the severity of the epidemic [37]. The findings may indicate that conservatives may experience more difficulty finding sources of COVID-19 vaccine information that they trust, thus highlighting the need to educate conservative media about COVID-19 vaccines. 

Finally, state health departments were frequently rated as trustworthy sources of COVID-19 information. Although state health departments often have websites, they may not have the resources to actively disseminate COVID-19 vaccine information. Strategies to disseminate COVID-19 information by health departments may also improve vaccine uptake. Although there were high ratings of trust in COVID-19 vaccine information from the CDC, the well-publicized political interference by the White House throughout the COVID-19 pandemic response could jeopardize its standing. Consequently, policymakers and the CDC itself should develop methods to insulate the agency from partisan politics that use the CDC for political gain. 

The findings expand the literature on trust and vaccine-related health behavior [54]. The results suggest that the dynamics of trust may encompass more than a unidirectional association where trust influences the probability of certain health behaviors. We found that trust in certain information sources was associated with encouraging or discouraging family and friends from being vaccinated. This finding suggests that trust may also lead to actions that may influence health-related social norms and affect others’ behaviors. In the current study, trust in sources of COVID-19 vaccine information was also linked to attitudes toward different groups and populations. There is ample evidence of medical distrust due to historical racism [55]. However, these findings expand that line of scholarship to show that individuals who exhibit high trust in conservative COVID-19 vaccine information sources tend to use race as an exclusionary category for those who should not be prioritized for vaccination, despite having higher rates of COVID-19 mortality. In contrast, they do not have the same view for older individuals who are also at high risk of COVID-19 mortality. 

Study limitations: The study response categories are subject to interpretation. Trust in sources of vaccine information was measured on a 4-point scale. A 10-point scale from “completely trust” to “no trust at all” may have provided more detailed information on levels of trustworthiness. Given the cross-sectional nature of the data, it is difficult to infer causality. Many of the behaviors, beliefs, and attitudes surrounding vaccination are likely to be reinforcing, and individuals tend to affiliate with similar others who may also reinforce their beliefs. One important question that this study cannot answer is whether, based on the correlation among vaccine information trust levels, a decline or increase in trust in one source may lead to a decline or increase in trust from other sources. In addition, this sample was not representative of the general population. However, even representative and national samples may miss respondents who do not trust survey researchers and further may have insufficient sample sizes for sub-analyses of minority populations. We did not examine other possible sources of COVID-19 vaccine information, and there may also be social desirability biases in the self-reports of attitudes and intentions. 

## 7. Conclusions

Future research should identify additional sources of trusted COVID-19 vaccine information and examine how social media may serve as a source of trust and distrust and lead people to trust/distrust traditional sources of health information. Research should also examine in greater detail how levels of trust influence not only one’s own behavior but also individuals’ encouragement and discouragement of others to be vaccinated. Additionally, the finding of a relationship between trust in sources of vaccine information and endorsement of policies to increase access to vaccines among vulnerable populations warrants further study. Finally, trust in sources of general health information could be assessed to examine whether these sources differ from vaccine-specific health information.

Taken together, study findings highlight how trust in sources of vaccine information influences vaccine perceptions and intentions for both self and others. The data also suggest that most people have high trust in several sources of COVID-19 vaccine information, and hence there are multiple avenues for disseminating information. Informational trust, however, is only one dimension of vaccine acceptance and uptake. Fair, equitable, and transparent access and distribution will also be essential to ensure trust in public health systems’ abilities to serve the population. 

## Figures and Tables

**Table 1:** Sociodemographic characteristics of the study population for a national survey on trusted sources of COVID-19 vaccine information in the US, 2020 (*N* = 586).

Background Variables	% Mean (SD)
Political Party Affiliation	
Democrat	45.7
Republican	21.2
Independent	29.0
Other	4.1
Race/Ethnicity	
White	81.1
Black	6.3
Asian	6.7
Other	6.0
Age in years	39.6 (11.7)
Household income greater than $60,000	45.6
Bachelor’s degree or higher	55.7
Female	57.3

**Table 2:** Descriptive statistics on vaccine attitudes, behaviors, and news consumption frequency among study participants in the US, 2020 (*N* = 586).

Coronavirus Variables	Response Categories	%
A vaccine would prevent me from getting the coronavirus	Strongly agree/agree	60.9
	Neither agree/disagree	27.0
	Strongly disagree/disagree	12.1
I will discourage my friends from getting the coronavirus vaccine when it is available.	Strongly agree/agree	5.6
	Neither agree/disagree	9.0
	Strongly disagree/disagree	85.4
I will encourage my family members to get a coronavirus vaccine when it is available.	Strongly agree/agree	59.4
	Neither agree/disagree	21.5
	Strongly disagree/disagree	19.1
I am worried about having bad side effects if I got a coronavirus vaccine.	Strongly agree/agree	64.0
	Neither agree/disagree	13.5
	Strongly disagree/disagree	22.6
I am concerned that a coronavirus vaccine will not be effective.	Strongly agree/agree	47.1
	Neither agree/disagree	17.9
	Strongly disagree/disagree	35.0
I am concerned that short cuts have been taken with coronavirus because of political pressures.	Strongly agree/agree	56.8
	Neither agree/disagree	14.3
	Strongly disagree/disagree	28.8
More vulnerable people, such as the elderly, should have priority for a coronavirus vaccine.	Strongly agree/agree	86.9
	Neither agree/disagree	11.1
	Strongly disagree/disagree	2.1
Groups that have higher rates of coronavirus deaths should have priority for a coronavirus vaccine.	Strongly agree/agree	66.0
	Neither agree/disagree	22.2
	Strongly disagree/disagree	11.8
On average, how often do you watch, listen, or read news about the coronavirus? *	Multiple times an hour	3.8
	Every 1–2 h	9.9
	A couple of times a day	35.2
	Once a day	29.6
	Less than once a day	21.5

*—Missing 1 respondent.

**Table 3:** Factor analysis and percentage of high trust ratings for each informational source among study respondents in the US, 2020 (*N* = 585).

Survey Item: “How Much Do You Trust Information about the Vaccine for Coronavirus from the Following Sources:”	Loading		Percent High Trust
	Factor 1	Factor 2	
	“Trust in Mainstream Sources”	“Trust in Politically Conservative Sources”	
Your healthcare provider?	0.706		75.5
Anthony Fauci, Director of the National Institute of Allergy and Infectious Disease?	0.826		70.3
The CDC?	0.810		66.2
Johns Hopkins University?	0.807		78.0
CNN?	0.658		37.0
Your State Health Department?	0.785		67.9
Pharmaceutical or Drug Companies?	0.671		32.3
The U.S. Food and Drug Administration (FDA)?	0.764		50.2
Fox News?		0.833	8.9
The White House?		0.865	13.7

**Table 4:** Results from a fixed-effects ANOVA on trust in mainstream and politically conservative sources, using likelihood of getting the coronavirus vaccine as the criterion, conducted among study participants in the US, 2020 (*N* = 585).

Vaccine Trust Measure		Sum of Squares	df	Mean Square	F	*p*
Trust in mainstream sources	Between groups	577.40	2	288.70	71.20	0.00
	Within Groups	2359.93	582	4.055		
	Total	2937.33	584			
Trust in politically conservative sources	Between groups	0.05	2	0.03	0.29	0.75
	Within Groups	51.73	582	0.09		
	Total	51.78	584			

**Table 5:** Tukey post hoc comparisons of 8-item vaccine trust scores between vaccine intention groups among study participants in the US, 2020 (*N* = 585).

Measure	Comparison	Mean Difference	Std. Error	*p*	95% CI (95%)
Trust in mainstream sources	Undecided vs. Intend not to vaccinate	1.12	0.26	<0.001	(0.50, 1.74)
	Intend to vaccinate vs. Intend not to vaccinate	2.32	0.20	<0.001	(1.86, 2.79)
	Intend to vaccinate vs. undecided	1.20	0.23	<0.001	(0.66, 1.75)

**Table 6:** Multiple logistic regression models with binary outcome measures of trusted information sources among study participants in the US, 2020 (*N* = 585).

	Trust in Mainstream Sources (MS)		Trust in Politically Conservative Sources (PCS)		Trust in Dr. Anthony Fauci	
	OR (95% CI)	aOR (95% CI)	OR (95% CI)	aOR (95% CI)	OR (95% CI)	aOR (95% CI)
A vaccine would prevent me from getting the coronavirus.	**2.76** **(2.22, 3.43)**	**1.47** **(1.10, 1.96)**	0.85 (0.71, 1.01)	1.04 (0.78, 1.38)	**2.68** **(2.17, 3.31)**	**1.49** **(1.10, 2.03)**
I will discourage my friends from getting the coronavirus vaccine when it is available.	**0.36** **(0.28, 0.46)**	0.75 (0.56, 1.00)	**1.45** **(1.20, 1.74)**	**1.47** **(1.12, 1.91)**	**0.37** **(0.29, 0.46)**	**0.67** **(0.51, 0.89)**
I will encourage my family members to get a coronavirus vaccine when it is available.	**2.62** **(2.19, 3.13)**	**1.61** **(1.28, 2.04)**	**0.85** **(0.74, 0.99)**	1.00 (0.78, 1.29)	**2.56** **(2.14, 3.05)**	**1.68** **(1.31, 2.16)**
I am worried about having bad side effects if I got a coronavirus vaccine.	**0.60** **(0.52, 0.69)**	1.00 (0.79, 1.28)	1.04 (0.90, 1.22)	1.19 (0.92, 1.53)	**0.61** **(0.52, 0.73)**	0.96 (0.72, 1.27)
I am concerned that a coronavirus vaccine will not be effective.	**0.67** **(0.58, 0.77)**	0.97 (0.77, 1.22)	0.95 (0.82, 1.11)	0.98 (0.78, 1.24)	**0.70** **(0.60, 0.82)**	0.94 (0.73, 1.21)
I am concerned that short cuts have been taken with coronavirus because of political pressures.	**0.67** **(0.59, 0.76)**	0.88 (0.71, 1.11)	**0.83** **(0.72, 0.95)**	**0.67** **(0.53, 0.85)**	**0.75** **(0.65, 0.87)**	1.11 (0.86, 1.43)
More vulnerable people, such as the elderly, should have priority for a coronavirus vaccine.	**2.60** **(2.02, 3.34)**	**1.50** **(1.09, 2.05)**	0.83 (0.67, 1.04)	1.30 (0.95, 1.78)	**2.44** **(1.92, 3.09)**	1.32 (0.96, 1.82)
Groups that have higher rates of coronavirus deaths should have priority for a coronavirus vaccine.	**1.87** **(1.58, 2.22)**	**1.32** **(1.05, 1.64)**	**0.65** **(0.55, 0.77)**	**0.70** **(0.56, 0.88)**	**2.06** **(1.72, 2.46)**	**1.41** **(1.11, 1.79)**
On average, how often do you watch, listen, or read news about the coronavirus?	**1.29** **(1.10, 1.51)**	0.91 (0.75, 1.11)	1.07 (0.90, 1.27)	0.84 (0.68, 1.03)	**1.42** **(1.19, 1.70)**	1.15 (0.93, 1.44)
Political Affiliation (Ref: Republican)	REF	REF	REF	REF	REF	REF
Democrat	**2.98** **(2.12, 4.19)**	**2.82** **(1.59, 5.02)**	**0.20** **(0.13, 0.31)**	**0.11** **(0.06, 0.20)**	**3.84** **(2.58, 5.72)**	**4.23** **(2.25, 7.96)**
Independent	**0.66** **(0.46, 0.95)**	1.78 (0.98, 3.23)	0.94 (0.62, 1.41)	0.28 (0.17, 0.48)	0.80 (0.54, 1.17)	**3.10** **(1.66, 5.78)**
Libertarian or member of another political party	**0.35** **(0.14, 0.86)**	0.70 (0.22, 2.21)	2.07 (0.90, 4.76)	0.49 (0.19, 1.28)	0.58 (0.25, 1.33)	2.41 (0.79, 7.34)
Race (Ref: White)	REF	REF	REF	REF	REF	REF
Black	0.59 (0.30, 1.16)	1.15 (0.48, 2.74)	0.76 (0.34, 1.70)	1.04 (0.40, 2.68)	0.60 (0.30, 1.19)	0.73 (0.31, 1.75)
Asian	1.47 (0.75, 2.85)	0.91 (0.40, 2.11)	0.83 (0.38, 1.79)	0.95 (0.37, 2.45)	1.25 (0.59, 1.61)	1.03 (0.40, 2.66)
Other	1.77 (0.86, 3.62)	1.25 (0.55, 2.88)	0.82 (0.36, 1.85)	0.84 (0.35, 2.04)	**4.81** **(1.45, 15.9)**	**5.65** **(1.19, 26.9)**
Age	1.01 (0.99, 1.02)	1.02 (0.99, 1.03)	1.01 (0.99, 1.02)	0.99 (0.98, 1.02)	0.99 (0.98, 1.01)	0.99 (0.97, 1.01)
Annual household income >$60,000	1.28 (0.92, 1.78)	1.12 (0.72, 1.70)	1.22 (0.85, 1.77)	1.07 (0.68, 1.69)	1.24 (0.86, 1.77)	1.19 (0.73, 1.93)
Completed a bachelor’s degree or higher	**1.55** **(1.12, 2.16)**	1.12 (0.73, 1.71)	0.81 (0.56, 1.18)	0.85 (0.54, 1.34)	**1.53** **(1.07, 2.19)**	1.13 (0.70, 1.82)
Male gender	0.74 (0.54, 1.04)	0.77 (0.50, 1.19)	1.40 (0.97, 1.03)	0.82 (0.53, 1.29)	0.85 (0.59, 1.21)	0.64 (0.39, 1.06)

Bolded values represent statistically significant covariates.

**Table 7:** Multiple linear regression with continuous 8-item MS vaccine trust measure as the dependent variable (range 0–8, higher scores indicating greater trust in sources on vaccine information) among study participants in the US, 2020 (*N* = 585).

Survey Items	Β (95% CI)	aβ (95% CI)
A vaccine would prevent me from getting the coronavirus.	**0.90** *** (0.74, 1.06)	0.16 (−0.05, 0.37)
I will discourage my friends from getting the coronavirus vaccine when it is available.	−**0.93** *** (−1.11, −0.74)	−**0.23** * (−0.43, −0.03)
I will encourage my family members to get a coronavirus vaccine when it is available.	**0.87** *** (0.74, 0.99)	**0.36** *** (0.18, 0.54)
I am worried about having bad side effects if I got a coronavirus vaccine.	−**0.57** *** (−0.71, −0.43)	−0.10 (−0.28, 0.08)
I am concerned that a coronavirus vaccine will not be effective.	−**0.49** *** (−0.63, −0.34)	−0.11 (−0.28, 0.06)
I am concerned that short cuts have been taken with coronavirus because of political pressures.	−**0.44** *** (−0.57, −0.30)	−0.03 (−0.19, 0.14)
More vulnerable people, such as the elderly, should have priority for a coronavirus vaccine.	**0.98** *** (0.76, 1.19)	**0.36** ** (0.13, 0.59)
Groups that have higher rates of coronavirus deaths should have priority for a coronavirus vaccine.	**0.67** *** (0.51, 0.83)	**0.22** ** (0.06, 0.38)
On average, how often do you watch, listen, or read news about the coronavirus?	**0.35** *** (0.18, 0.52)	0.14 (−0.01, 0.29)
Political Affiliation (Ref: Republican)	REF	REF
Democrat	**1.20** *** (0.84, 1.55)	**0.60** ** (0.17, 1.03)
Independent	−**0.61** ** (−1.01, −0.21)	0.12 (−0.33, 0.56)
Libertarian or member or another political party	−**1.04** * (−1.95, −0.12)	−0.35 (−1.18, 0.47)
Race (Ref: White)	REF	REF
Black	−**0.76** * (−1.50, −0.01)	−0.24 (−0.89, 0.40)
Asian	−0.11 (−0.84, −0.62)	−0.34 (−0.97, 0.29)
Other	**0.77** * (0.01, 1.54)	0.24 (−0.41, 0.89)
Age	0.25 (−0.02, 0.51)	−0.00002 (−0.01, 0.01)
Annual household income >$60,000	0.29 (−0.08, 0.65)	0.11 (−0.21, 0.43)
Completed a bachelor’s degree or higher	0.46 * (0.10, 0.83)	0.08 (−0.24, 0.41)
Male gender	0.26 (−0.10, 0.63)	0.14 (−0.19, 0.46)

* *p* < 0.05; ** *p* < 0.01; *** *p* < 0.001.

## Data Availability

The data are available through the first author based on IRB approval.
